# Beyond Inhibition: Sublethal Rifampicin-Induced Molecular Adaptations Confer Phenotypic Drug Tolerance in Mycobacteria

**DOI:** 10.1021/acsinfecdis.5c00701

**Published:** 2026-04-28

**Authors:** Blake D. Stuart, Anja van der Merwe, Melissa D. Chengalroyen, Atica Moosa, Digby F. Warner, Jonathan M. Blackburn, Tariq A. Ganief

**Affiliations:** † Division of Chemical and Systems Biology, Department of Integrative Biomedical Sciences, Faculty of Health Sciences, 71985 University of Cape Town, Cape Town 7925, South Africa; ‡ Molecular Mycobacteriology Research Unit, Division of Medical Microbiology, Department of Pathology, Faculty of Health Sciences, University of Cape Town, Cape Town 7925, South Africa; § Institute of Infectious Disease and Molecular Medicine, Faculty of Health Sciences, University of Cape Town, Cape Town 7925, South Africa

**Keywords:** Mycobacterium tuberculosis, Mycobacterium smegmatis, phenotypic tolerance, sublethal rifampicin, proteomics, mass spectrometry

## Abstract

Tuberculosis (TB) remains a major global health threat, largely due to *Mycobacterium tuberculosis* (Mtb) resilience, which can be exacerbated by exposure to sublethal antibiotic concentrations arising from factors such as patient nonadherence and the granuloma structure which limits drug penetration. Within host granulomas, Mtb can exhibit both phenotypic tolerance and genotypic resistance, complicating curative treatments. This study aimed to determine whether sublethal rifampicin acts as a signaling molecule in *Mycobacterium smegmatis* (Msm) and the attenuated Mtb H37Ra strain, triggering phenotypic changes that promote tolerance to lethal drug levels. Msm exposed to half-MIC rifampicin showed an initial transient growth deceleration followed by a resumption of proliferation, indicating the acquisition of phenotypic tolerance. Deep data-independent acquisition (DIA) mass spectrometry-based proteomic profiling revealed that the early response (45 min) involved the upregulation of ribosomal proteins, DNA replication, and de novo purine biosynthesis. Proteins associated with phenotypic resistance (e.g., RpoZ, GidB, WhiB2) and efflux transporters were also upregulated. As Msm recovered (180 min), its proteome largely returned to baseline, but key resistance-associated pathways, including the Rifampin ADP-ribosyltransferase superfamily and certain efflux systems, remained dysregulated. Parallel studies on Mtb H37Ra also demonstrated a distinct proteomic shift, comprising conserved adaptive responses such as ribosomal perturbation and compensatory transcriptional activity, as well as species-specific dysregulation of drug influx/efflux pumps and cell envelope remodelling via the polyketide synthase family. These findings demonstrate that sublethal rifampicin exposure primes mycobacteria for enhanced tolerance to lethal drug concentrations, underscoring a significant challenge in current TB therapy.

## Graphical abstract

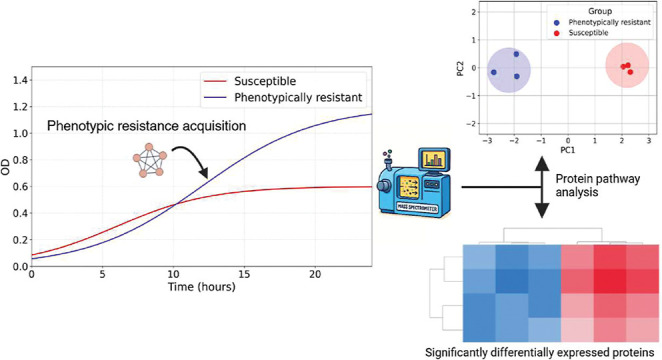

## Introduction

Tuberculosis (TB), caused by infection with *Mycobacterium tuberculosis* (Mtb), remains a major global health threat^1^ despite advances in diagnostics and treatment, such as the Xpert MTB/RIF assay^2^ and shorter drug regimens. A key challenge in TB control is Mtb’s resilience against a broad range of antimicrobial agents.^3,4^ This is partly driven by frequent exposure to sublethal antibiotic concentrations—arising due to factors such as patient nonadherence and inconsistent dosing^5^ allowing bacteria to survive, adapt, and develop resistance under low-dose drug selective pressure.

A defining feature of TB infection is the formation of granulomas,^6^ immune-constructed structures that encapsulate Mtb. These granulomas create hypoxic, nutrient-limited environments that restrict drug penetration, due in-part to poor vascularization.^7^ Within these niches, Mtb can exhibit both phenotypic tolerance and genotypic resistance, increasing its survival under fluctuating antibiotic levels. Similarly, any phenotypic tolerance could further facilitate genotypic resistance generation.^8^ Therefore, understanding the Mtb molecular response to sublethal antibiotic exposure is crucial for refining treatment strategies and potentially curbing the rise of multidrug-resistant (MDR) and extensively drug-resistant (XDR) TB strains.

As the frontline anti-Mtb drug^9^ and the most frequently identified target of genotypic resistance, understanding rifampicin induced molecular signaling, facilitating tolerance is vitally important. Our group has previously shown that sublethal rifampicin induces phenotypic tolerance, distinct from genotypic resistance development, which is underpinned by distinct responses including activation of the SOS response and ABC transporter modulation,^10^ differing from responses triggered by lethal antibiotic doses. While *Mycobacterium smegmatis* (Msm) is a genetically similar and widely used model for Mtb, it lacks some pathogenic features, including the limited capacity to survive within macrophages.^11^

Here, we aimed to determine the molecular signaling in Msm induced by sublethal rifampicin concentrations and to identify phenotypic changes that may promote tolerance to subsequent lethal levels of drug. We monitored Msm growth under sub-MIC rifampicin conditions and used deep DIA-PASEF based proteomics to identify underlying molecular adaptations. We then validated these findings in Mtb H37Ra, a well-characterized attenuated strain,^12^ to assess both conserved and divergent responses between the two species.

To profile these proteomic changes, we employed data-independent acquisition (DIA) mass spectrometry, which offers deep proteomic coverage^13^ and is uniquely positioned to characterize complex cellular signaling in this model. This approach enabled detailed tracking of protein expression patterns in response to sublethal rifampicin exposure, providing insights into the regulatory pathways and protein networks involved in adaptation, virulence, and drug phenotypic resistance development. This understanding may lead to the development of novel adjunctive drug targets which may shorten treatment times and decrease resistance development.

## Results

### Mycobacteria Acquire Phenotypic Rifampicin Tolerance within One Doubling Cycle

Msm cultures were treated with rifampicin at a subinhibitory concentration of 3.9 μg/mL (halfxMIC), determined from MIC assays of both wild-type (WT) and the PhoP CRISPRi knockdown strain, which each yielded a MIC value of 7.8 μg/mL (Supporting Information Figure S1). In the absence of anhydrotetracycline (ATc), the knockdown strain behaves identically to WT in terms of growth kinetics and rifampicin susceptibility (Supporting Information Figure S2). As such, the noninduced condition (−ATc) was used as a WT reference for downstream proteomic analyses.

Cultures were monitored over 8 h (Figure 1). Initially, rifampicin-treated samples (halfxMIC) mirrored untreated controls (0xMIC) in early exponential phase. A transient deceleration in growth occurred between 120 and 180 min post-treatment, followed by resumption of proliferation that became indistinguishable from 0xMIC cultures (Figure 1A). Comparable trends have been reported in previous work.^10,14,15^ Growth rates echo this trajectory (Figure 1B), with halfxMIC cultures showing a brief decline, then recovering to match and eventually surpass 0xMIC samples.

**Figure 1:**
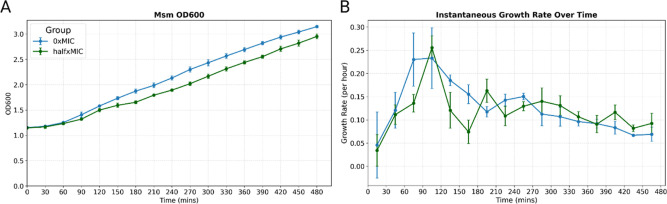
Growth kinetics of Msm in response to sublethal rifampicin. (A) HalfxMIC Msm experiences a modest growth arrest between ∼90 and 180 min after which it resumes exponential growth mirroring 0xMIC cultures. (B) Growth rate estimates based on the growth kinetics similarly exhibit a sharp decline in growth rate prior to adaptation and increased growth rate at ∼180 min.

To understand the molecular mechanisms of the rifampicin induced arrest and the associated phenotypic recovery, proteomic analysis was performed at 45 (T1) and 180 (T2) minutes post-treatment—time points that encompass the inflection zone and capture relevant signaling shifts (Supporting Information Figure S3).

### Proteomic Analysis Offers Deeper Understanding of Rifampicin Tolerance

A deep DIA-PASEF proteomic analysis using the tims-TOF Pro2 identified and quantified 23,256 peptides from 3618 proteins. After data filtering and outlier removal, the relative abundance of proteins between samples was consistent (Figure 2A). At T1, 0xMIC and halfxMIC groups separate distinctly from each other as well as from T2 groups, whereas at T2, replicates cluster tightly, irrespective of treatment concentration, showing that the rifampicin induced response decreases over time (Figure 2B). This sample clustering and consistency was further seen using hierarchical clustering which showed that, although distinct, T2_halfxMIC expression patterns resemble T2_0xMIC with functional differences being maintained (Figure 2C).

**Figure 2:**
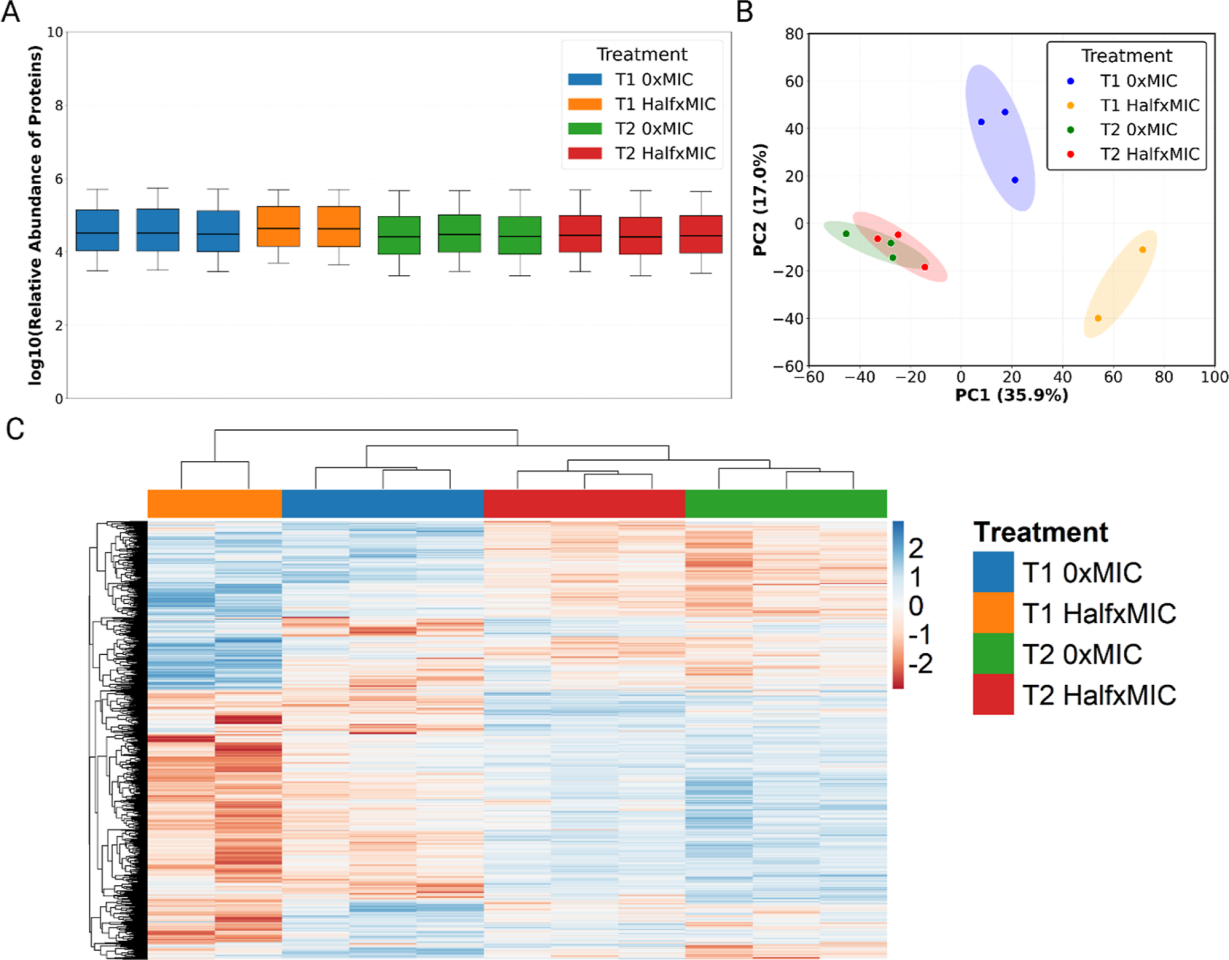
Proteomic overview of Msm’s response to sublethal rifampicin. (A) Even distribution of protein intensities across all samples suggest reproducible quantitation across the experiment. (B) T1 0xMIC and halfxMIC treated sample clusters are entire distinct suggesting highly reproducible differential protein expression patterns. Conversely, T2 0xMIC and halfxMIC samples cluster more closely together but distinctly from T1 samples. This suggest a return to baseline in the expression patterns despite rifampicin being present. (C) Differentially expressed proteins highlight the distinct but highly reproducible expression patterns of rifampicin treated Msm over time. While early rifampicin-induced differential expression wanes over time, many clusters are maintained.

Limma differential expression analysis detected 940 proteins significantly differentially expressed between T1_0xMIC and T1_halfxMIC and 1290 proteins significantly differentially expressed between T1_halfxMIC and T2_halfxMIC. Of these, 660 were uniquely dysregulated in the halfxMIC condition (unique in that the list excludes proteins that were significantly differentially expressed between T1_0xMIC and T2_0xMIC). At T2, only 329 proteins were significantly differentially expressed between T2_0xMIC and T2_halfxMIC, further suggesting that the response to rifampicin wanes over time despite maintained rifampicin presence (data not shown).

### Initial Rifampicin Exposure is Characterized by Upregulation of Translation and Downregulation of Transcription and de Novo IMP Biosynthesis

At T1, a tightly clustered group of ribosomal proteins, involved in translation, gene expression and macromolecule biosynthetic processes were upregulated in the halfxMIC compared to 0xMIC samples (Figure 3A). Conversely, a number of pathways were downregulated in the T1_halfxMIC compared to T1_0xMIC, including RNA polymerase (Figure 3Bi), DNA templated DNA replication (Figure 3Bii), *t*-RNA aminoacylation for protein translation (Figure 3Biii), purine metabolism (Figure 3Biv) and de novo IMP biosynthesis (Figure 3Bv).

**Figure 3:**
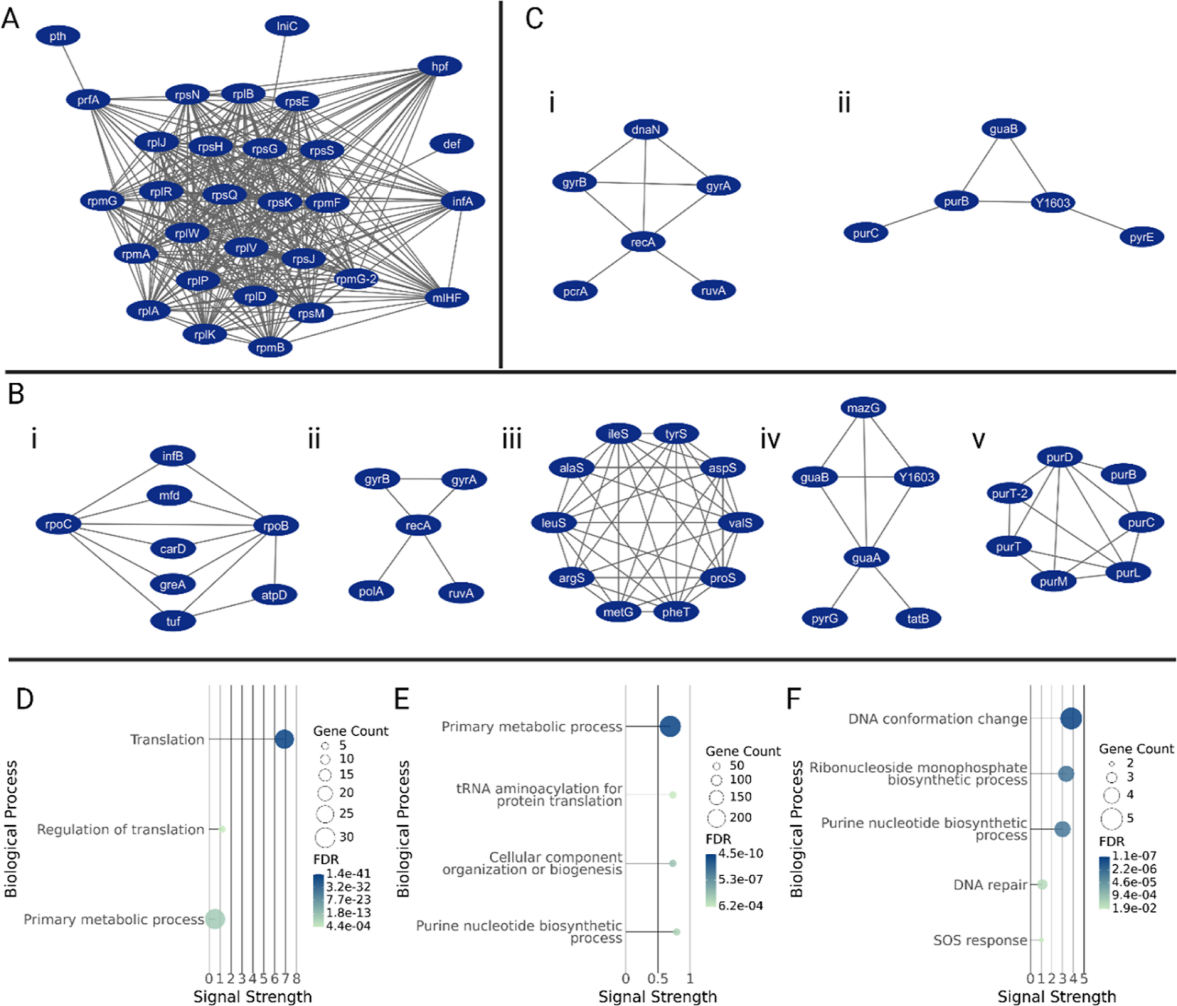
Functional proteomic adaptations in Msm to sublethal rifampicin. (A,D) Cluster of ribosomal proteins and associated GO biological functions significantly upregulated in T1_halfxMIC compared to T1_0xMIC, indicating increased translational activity in response to rifampicin. (B,E) Protein clusters and associated biological functions significantly downregulated in T1_halfxMIC compared to T1_0xMIC. Functions include those involved in RNA polymerase (i), DNA replication (ii), *t*-RNA aminoacylation (iii), purine metabolism (iv) and de novo IMP biosynthesis (v). (C,F) Clusters of significantly upregulated proteins over time (in T2_halfxMIC compared to T1_halfxMIC) and their associated biological functions in response to rifampicin, which include DNA replication and repair (i) and de novo purine biosynthesis (ii).

Within these dysregulated clusters, we observed upregulation of several individual proteins including RpoZ, GidB, RdgB, WhiB, WhiB2, ClgR, Phd, Hup and Lsr2 which are known to play a role in phenotypic resistance acquisition^16–23^ as well as upregulation of helix-turn-helix (HTH) multiple antibiotic resistance (Mar) proteins regulated by MSMEG_2538 and MSMEG_5688. In addition, we observed significant dysregulation of MSMEG_6509, a Mar ABC transporter that, unlike other Mar transporters, has previously been proposed to play a role in rifampicin import.^24^ MSMEG_6509 was initially upregulated at T1, before being depleted in halfxMIC cultures, potentially contributing to late stage resistance acquisition.

Despite the general upregulation of ribosomal proteins and protein translation clusters, we observed significant downregulation of RpoB, the target of rifampicin, at T1. This is likely as a result of the general stress induced arrest of transcription due to the upregulated helix-turn-helix gluconate operon transcriptional repressor proteins. This is consistent with previous reports that rifampicin induces a rapid decrease in RpoB transcript expression followed by an increase due to promoter inhibition and switching induced by rifampicin.^19^ Notably, given the reported substantial uncoupling of mycobacterial transcriptomes and proteomes,^25,26^ the present data now provides an accurate quantitative description of the magnitude of this dysregulation at the protein level.

### The Change in Protein Expression over Time in Response to Rifampicin Revealed Upregulation of Transcription, de Novo IMP Biosynthesis and SOS Response, as Well as Downregulation of Translation

Comparing halfxMIC cultures at T1 and T2, several functional protein clusters including DNA replication, de novo purine biosynthesis and SOS response pathway were upregulated in T2_halfxMIC compared to T1_halfxMIC (Figure 3C i–ii). In addition, the cluster of ribosomal proteins that were upregulated at T1 (Figure 3A) were now observed to be downregulated at T2.

The upregulation of DNA replication is driven by RpoC, InfB, CarD and LepA and the SOS response—RecA, RuvA, DnaN and DnaG. MtrA, part of the MtrAB two-component regulatory system, was also upregulated. The mtrAB system has been shown to be essential for both in vitro and in vivo survival of Mtb.^27,28^ MtrA expression levels have also been shown to affect drug resistance and cell morphology of Msm.^29^ After 180 min, RpoB expression in halfxMIC cultures returned to levels matching 0xMIC controls and we discerned a decrease in the resistance associated proteins previously upregulated, such as WhiB2, ClgR and RbpA.

### Diminished Dysregulation at T2 Reveals Adaptive Resilience to Rifampicin Stress

At T2, the number of dysregulated proteins and pathways between 0xMIC and halfxMIC were markedly reduced compared to between 0xMIC and halfxMIC at T1. This trend may suggest that phenotypic tolerance mechanisms were acquired, enabling the cells to adapt and alleviate antibiotic-induced stress, even in the continued presence of rifampicin. Consequently, the proteomic profile of halfxMIC cultures at T2 resembled that of 0xMIC controls. Importantly though, while many cellular functions appear restored, key processes remain dysregulated. Among these, we observed sustained upregulation, in halfxMIC compared to 0xMIC, of the Rifampin ADP-ribosyltransferase (Arr) superfamily, which contains a rifampicin resistance protein intrinsic to Msm. Additional upregulated pathways include HTH MAR proteins, small multidrug resistance (SMR) protein pathways and DNA-directed RNA polymerases—RpoA in particular. Oxidoreductase activity is also upregulated, in halfxMIC compared to 0xMIC, at T2, with notable contributors such as KatG3, MsrB and an ABC transporter with predicted efflux activity. Conversely, several pathways are significantly downregulated at T2, in halfxMIC compared to 0xMIC, including amino acid ABC-transporters, virulence Mce family proteins, and LpqB - the latter is known to modulate activity of the MtrAB system which has a role in antibiotic resistance acquisition and cell morphology changes^29^ as well as MSMEG_6509, which is implicated in rifampicin import.^24^ At T2, the proteomic profile of the halfxMIC samples begins to resemble that of the untreated control, suggesting an initial recovery from rifampicin stress. However, several resistance- and stress-associated pathways remain persistently dysregulated, indicating that while global protein expression trends normalize, key regulatory modules continue to reflect long-term adaptation to ongoing drug pressure.

### Sub-MIC Rifampicin Treatment Generates a Protective Molecular Phenotype toward Antibiotic Induced Stress

Cultures pretreated with a sub-MIC dose of 1.5 μg/mL rifampicin exhibited a marked growth advantage upon subsequent addition of 7.8 μg/mL (1xMIC) rifampicin (Figure 4). In particular, pretreated samples entered exponential growth in the presence of 1xMIC antibiotic challenge, whereas nonpre-treated cultures failed to adapt and showed minimal growth under 1xMIC conditions. Notably, both pretreated and nonpre-treated cultures that were subsequently treated with a DMSO vehicle-only control and grew similarly, confirming that pretreatment did not compromise cell viability. These results demonstrate that sub-MIC rifampicin exposure primes Msm for enhanced tolerance to higher drug concentrations, reflecting a protective molecular phenotype. To rule out survival due to the selection of genotypic mutants, ∼1.1 × 10^7^ pretreated cells were plated onto 10xMIC rifampicin solid media. After 4 days of incubation, only a single colony was observed (Supporting Information Figure S4).

**Figure 4:**
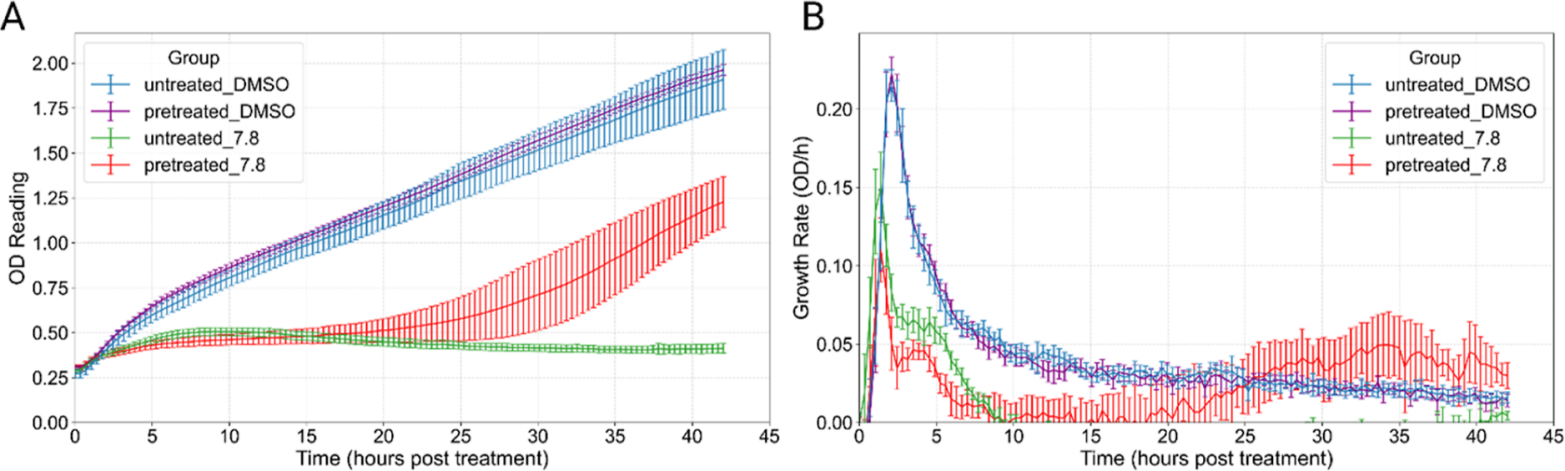
Phenotypic drug tolerance acquisition in Msm pretreated with sub-MIC Rifampicin. (A) OD600 readings over a 42 h period for Msm cultures shows samples pretreated with 1.5 μg/mL rifampicin for 7 h and subsequently challenged with a lethal dose of 7.8 μg/mL (1xMIC) rifampicin, compared to nonpre-treated controls and DMSO controls. Notably, pretreated samples exhibited a marked growth advantage and entered exponential growth despite the lethal antibiotic challenge, whereas nonpre-treated cultures showed no growth. (B) Growth rate estimates show that after 19 h, pretreated Msm experiences a dramatic growth rate increase despite lethal rifampicin doses compared to the nonpre-treated culture showing no significant growth.

### Sublethal Rifampicin-Induced Proteomic Changes are Also Observed in Mtb H37Ra

Although Msm is a well-accepted surrogate model of Mtb, we sought to validate these observations using the attenuated Mtb H37Ra applying a comparable DIA-PASEF proteomics workflow. To assess the proteomic responses to sublethal rifampicin exposure we first determined the minimum inhibitory concentration (MIC) to be 0.0012 μg/mL (Supporting Information Table S1), with 0.0006 μg/mL selected as the halfxMIC concentration for subsequent analyses. Given the previously observed rapid antibiotic-induced signaling, we focused on capturing early proteomic adaptations. Accordingly, samples were collected 2 h post-treatment to characterize early rifampicin induced molecular responses. This analysis identified and quantified a total of 2523 proteins, of which 556 were significantly dysregulated between 0xMIC and halfxMIC groups. Of these, 323 proteins were downregulated and 233 upregulated, indicating substantial proteomic rearrangement in response to sublethal rifampicin exposure.

### Distinct Proteomic Remodelling in Mtb H37Ra Induced by Sub-MIC Rifampicin

Upon exposure to halfxMIC rifampicin, Mtb H37Ra exhibited reproducible sample intensity distributions (Figure 5A) and a markedly distinct and reproducible proteomic response compared to 0xMIC controls (Figure 5B) indicative of a highly specific and consistent response across replicates. In contrast, 0xMIC samples displayed greater variance, likely reflecting stochastic fluctuations in proteome composition under nonstressed conditions. Hierarchical clustering (Figure 5C) reinforces this observation, with halfxMIC samples grouping discretely from controls, underscoring the impact of rifampicin-induced stress on cellular remodelling at the proteomic level.

**Figure 5:**
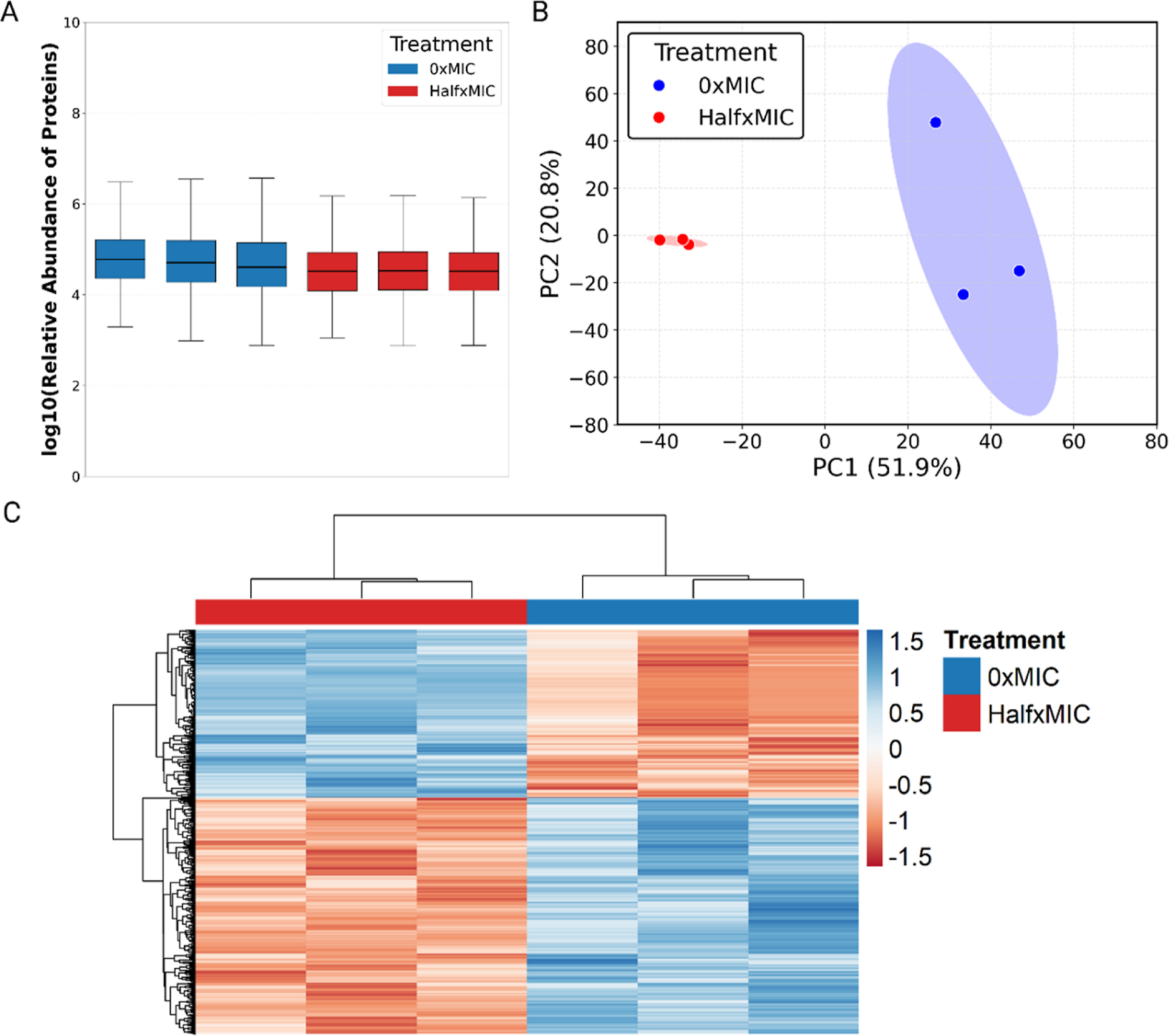
Overview of the Mtb H37Ra proteomic response to halfxMIC rifampicin treatment. (A) Even distribution of protein intensities across all samples suggest reproducible quantitation across the experiment. (B) The distinct separation between 0xMIC and halfxMIC treated samples, indicating a highly specific and reproducible proteomic response induced by rifampicin. (C) The clustering of the differentially expressed proteins highlights the extent and reproducibility of proteomic rearrangement in response to sublethal rifampicin exposure.

HalfxMIC rifampicin treatment in Mtb H37Ra triggered a multifaceted proteomic response with distinct functional clusters of differentially regulated proteins emerging across key biological processes, many of which were congruent with the response to halfxMIC rifampicin described above for Msm, including: a prominent downregulation of ribosomal proteins indicating translation repression and suggesting a conserved response to rifampicin challenge (Figure 6A); upregulation of DNA-directed RNA polymerases, RpoA and RpoC; and enriched GO terms related to transcriptional activity and protein complex formation, collectively suggesting a compensatory transcriptional response to RpoB inhibition that is shared with Msm.

**Figure 6:**
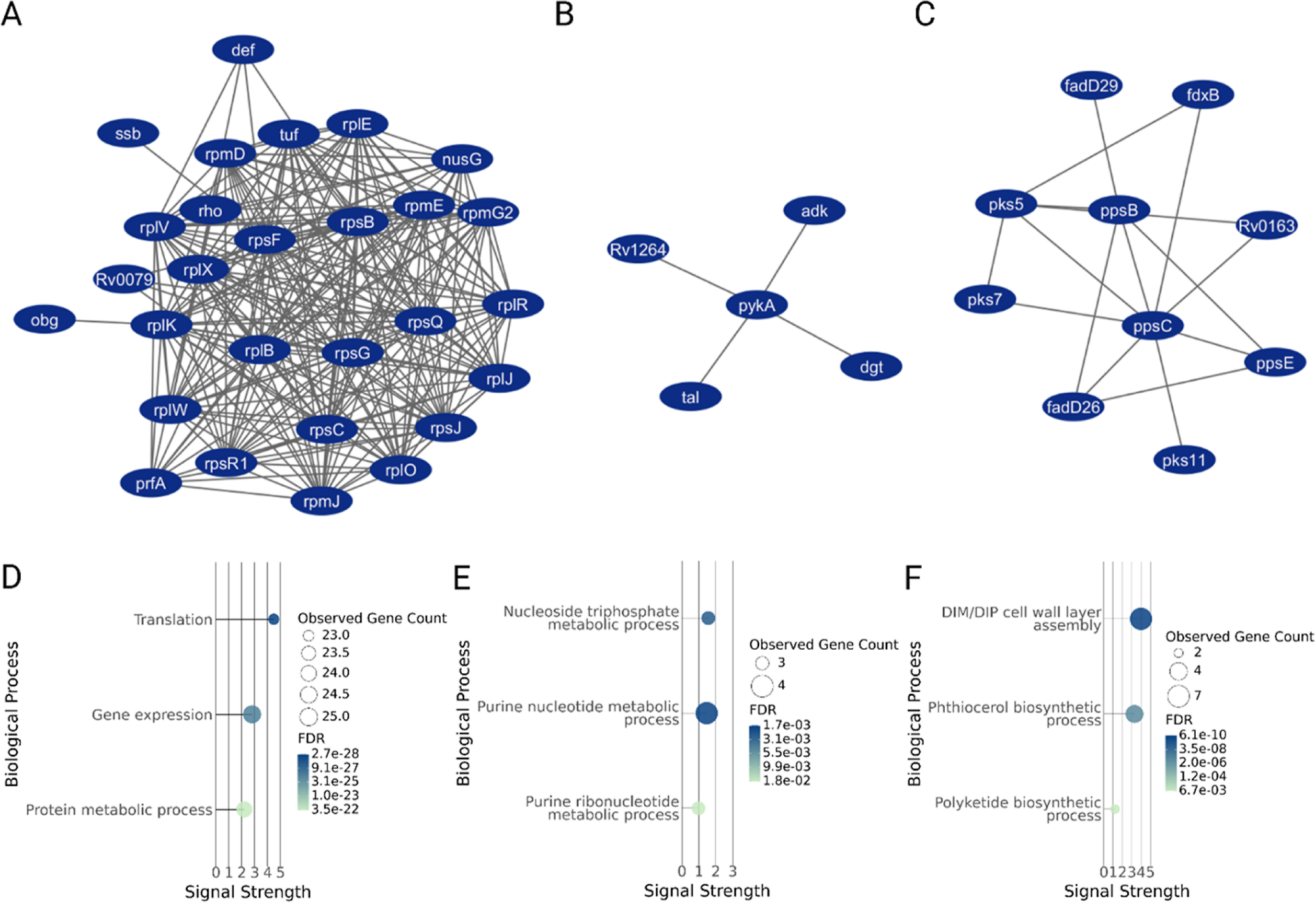
Functional proteomic remodelling in Mtb H37Ra by sub-MIC rifampicin. (A,D) Cluster of significantly altered proteins and corresponding GO enrichment analysis for biological processes. This cluster shows a prominent downregulation of ribosomal proteins, indicating translation repression, which is a conserved translational response to rifampicin stress. (B,E) The cluster of significantly differentially regulated proteins and associated GO biological process enrichment analysis includes the downregulation of proteins linked to purine metabolism (e.g., nucleoside triphosphate and purine nucleotide biosynthesis), consistent with an acute stress response. (C,F) Cluster of significantly altered proteins and corresponding GO enrichment analysis for biological processes. This cluster shows robust activation of lipid biosynthetic pathways, including strong upregulation of several members of the polyketide synthase (PKS) family (e.g., Pks5, Pks7, Pks11) and associated polyketide synthesis complex components (PpsE, PpsC, PpsB). This indicates active remodelling of the cell envelope, a strategy to potentially reduce antibiotic permeability.

The downregulation of proteins linked to purine metabolism seen in the Msm data was also observed in Mtb H37Ra (Figure 6B). This included pathways such as nucleoside triphosphate and purine nucleotide biosynthesis, which indicates a shift in cellular metabolism toward a lower energy state, consistent with an acute stress response. Notably though, a unique and robust activation of lipid biosynthetic pathways was observed in Mtb H37Ra and not in Msm. In more detail, GO enrichment analysis indicates increased biosynthesis and assembly of complex lipids, suggesting that Mtb H37Ra actively remodels its cell envelope in response to rifampicin stress. This inference of altered lipid biosynthesis is based on the strong upregulation of several members of the polyketide synthase (PKS) family (Figure 6C,F), including Pks5, Pks7, and Pks11 and associated polyketide synthesis complex components PpsE, PpsC and PpsB, implying a coordinated attempt to reduce antibiotic permeability^30^ and representing a distinct facet of Mtb H37Ra’s rifampicin response compared to Msm.

## Discussion

Tuberculosis (TB), caused by Mtb, remains a profound global health challenge despite advancements in diagnostics and treatment. A critical aspect of Mtb’s persistence and the challenge in TB control lies in its remarkable resilience to antimicrobial agents, particularly under exposure to sublethal antibiotic concentrations. Such exposure, often due to factors such as patient nonadherence and loss of vascularization at the site of disease,^31^ enables bacteria to survive, adapt, and develop resistance. Notably, within the host granuloma hypoxic and nutrient-limited environment, Mtb has the potential to exhibit phenotypic tolerance and/or genotypic resistance, complicating curative treatment outcomes.^32^ Understanding the molecular adaptations triggered by transient sublethal antibiotic exposure is therefore key to refining treatment strategies and curbing the rise of multidrug- (MDR) and extensively drug-resistant (XDR) strains. The present study therefore understand the phenotypic adaptions to sublethal rifampicin observed in Msm and Mtb H37Ra as they may facilitate the development of genetic resistance.

Our deep DIA-based proteomic analysis revealed that sub-MIC rifampicin elicits distinct adaptive responses in both Msm and Mtb H37Ra. In Msm, this dynamic program was marked by the upregulation of translation alongside the downregulation of pathways related to RNA polymerase, DNA replication, and de novo purine biosynthesis, reflecting an early stress response that prioritizes rapid protein synthesis even as general transcription is transiently suppressed. Notably, GidB—a regulator of translation fidelity—emerged as a key player through its methylation of 16S rRNA, modulating mistranslation rates^33^ that have been shown to be both necessary and sufficient for phenotypic resistance acquisition in mycobacteria.^34,35^ As growth resumed, resistance-associated pathways declined, yet tolerance markers such as HTH MAR proteins,^4,36^ Rifampin ADP-ribosyltransferase,^37,38^ SMR proteins, and efflux-related ABC transporters remained upregulated, indicating a persistent molecular signature of drug tolerance. Phenotypically, this was reflected by a transient deceleration in growth followed by re-entry into exponential proliferation, with experimental validation showing that pre-exposure to sub-MIC rifampicin conferred a significant growth advantage under subsequent lethal challenge, consistent with a preconditioning effect that promotes tolerance. Importantly, the survival we observed cannot be explained by the selection of rifampicin-resistant mutants. The number of survivors far exceeded the expected spontaneous mutation frequency, and pretreated cells failed to grow robustly on 10xMIC rifampicin plates.

Our proteomic experiments in Msm were conducted in an uninduced phoP CRISPRi background, which behaved indistinguishably from the wild-type parent in terms of growth and rifampicin susceptibility. The consistency of our results with previous studies performed in true WT Msm further supports that the likelihood of basal PhoP silencing affecting our findings is minimal.

The distinct response observed in Mtb H37Ra is not unexpected given its distinct taxonomy and reduced gene repertoire relative to Msm. Among the notable differences was the downregulation of ribosomal proteins at 2 h, which may represent a transient suppression before stabilizing at later time points, in contrast to the early upregulation and subsequent normalization of translation machinery in Msm. More striking, however, were the divergent adaptive strategies: while ABC transporter dysregulation was prominent in Msm, it was absent in Mtb H37Ra, which instead uniquely upregulated the polyketide synthase (PKS) family involved in phthiocerol dimycocerosate (PDIM) synthesis, suggesting active remodelling of the cell envelope to reduce antibiotic permeability. Mtb H37Ra also showed upregulation of DNA-directed RNA polymerase, consistent with a compensatory transcriptional response to RpoB inhibition that mirrors aspects of the Msm program. Together, these findings demonstrate that sublethal rifampicin induces a reproducible proteomic shift within 2 h of exposure in Mtb H37Ra, revealing both conserved features—such as perturbation of ribosomal machinery—and species-specific differences that likely carry physiological consequences in TB disease.

To our knowledge, this study represents one of the first deep-proteomic investigations into the early adaptive responses of Mtb H37Ra to sublethal rifampicin. By comparing these results with our Msm data, we identified an evolutionarily conserved translational “stress signature” shared across species, alongside divergent strategies such as the unique upregulation of the polyketide synthase (PKS) family in Mtb H37Ra. Although both our previous work and independent studies have examined proteomic responses to rifampicin, the present study specifically focuses on the early effects of sublethal rifampicin exposure on the proteome. For example, de Keijzer et al. (2016) investigated high-dose rifampicin exposure in Mtb and proposed DosR-initiated dormancy as a survival strategy under prolonged drug stress.^39^ In contrast, our data reveal that even short-term, low-dose rifampicin exposure elicits a range of early metabolic adaptations. Among these, the downregulation of purine metabolism pathways in both Msm and Mtb H37Ra is particularly relevant, as it suggests a shift toward reduced energy demand consistent with conditions known to activate the DosR regulon.^40^ While DosR is classically triggered by hypoxia and redox stress, suppression of nucleotide biosynthesis may contribute to a comparable low-energy signaling environment, thereby facilitating DosR-mediated adaptive responses. Consistent with this interpretation, we previously demonstrated that DosR (DevR) was dysregulated in Msm following ∼6 h of sublethal rifampicin exposure.^10^

These divergent adaptations are likely a result of the different natural environments to which these species have evolved. Msm, as a soil microbe, may frequently encounter a broad range of chemical insults, favoring adaptations such as broad-spectrum efflux pumps and general stress responses to survive diverse toxins. Conversely, Mtb, as a human-adapted pathogen, thrives within the complex host environment, particularly within granulomas where it must contend with immune pressures and variable drug penetration. In this context, modifying or reinforcing the cell envelope lipids provides a robust strategy for Mtb to resist both host defenses and antibiotic permeation, a more specific and perhaps more energetically efficient adaptation for its unique niche. This highlights that while Msm serves as a valuable model, its utility may be limited for understanding all phenotypic drug resistance mechanisms critical to TB disease.

In conclusion, our study provides important new insights into the diverse and complex adaptive strategies employed by mycobacteria in response to sublethal rifampicin exposure. The rapid and dynamic proteomic rearrangements observed in both Msm and Mtb H37Ra underscore how even limited exposure to sublethal drug concentrations can act as powerful signaling molecules, promoting the acquisition of phenotypic tolerance to subsequent lethal doses. These adaptive mechanisms involve a complex interplay of transcriptional and translational reprogramming, cell envelope remodelling, and metabolic shifts. The ability of mycobacteria to phenotypically adapt and tolerate antibiotics under conditions relevant to TB infection in a relatively short time frame highlights a significant challenge in current TB therapy. These phenotypic adaptations would likely facilitate the subsequent development of genetically resistant strains. Further understanding these responses may guide drug development aimed at preventing drug resistance development or enhance drug sensitization.

## Materials and Methods

### Mycobacterial Culture

*M. smegmatis* (Msm) mc^2^155 (also referred to as Msm WT) and *M. tuberculosis* (Mtb) H37Ra were used in this study. A PhoP CRISPRi hypomorph strain was generated from Msm mc^2^ 155 using an optimized Msm CRISPRi backbone (Addgene—Plasmid#163635) containing a kanamycin resistance gene and ATC inducible tetracycline operator.^41^ The hypomorph was created as previously described.^42^ Cultures were grown in 7H9 Middlebrook (Difco) enriched with 10% oleic acid-albumin-dextrose-catalase (OADC), 0.2% glycerol and 0.05% Tween-80 at 37 °C with the Msm cultures additionally containing 50 μg/mL kanamycin. The minimum inhibitory concentrations (MIC) of rifampicin was determined using a microbroth dilution assay and by measuring the optical density (OD) A600 and fluorescence on a FLUOstar plate reader (BMG LabTec), with excitation and emission of 544 and 590 nm, respectively. Rifampicin (Merck) was prepared and diluted in dimethyl sulfoxide (DMSO). The MIC’s were determined based on treatment curves showing the % inhibition against concentration, generated from the fluorescence data.

### Growth Kinetics

As we have previously shown that rifampicin induces a brief growth arrest followed by resumption of exponential growth,^15^ we generated growth curves for Msm using 0 and halfxMIC (3.9 μg/mL) rifampicin. Briefly, OD600 measurements were taken every 30 min over 8 h.

Growth curves were conducted in order to determine the ideal time points for proteomic extraction in order to capture the desired proteomic signaling. Cells were grown to an OD of ∼1.0–1.2, and readings were taken every 30 min using a spectrophotometer (WPA). For each experiment, duplicates were included for + and - ATc along with three biological replicates for each sample.

To validate the hypothesis that, when exposed to sublethal doses of rifampicin, Msm adapts its molecular makeup enabling it to tolerate otherwise lethal doses of rifampicin, Pioreactors (Pioreactor Inc.) were used to generate growth curves. Msm WT mc^2^ 155 was grown in Erlenmeyer flasks under the same media and growth conditions as utilized previously. Two cultures of OD 0.25 were treated with DMSO (1%) or 1.5 μg/mL rifampicin respectively, and allowed to grow for 7 h. From these batch cultures, three replicates of each treatment condition was prepared at OD 0.25 in 20 mL Pioreactor vials; DMSO—DMSO, DMSO—7.8 μg/mL rif, 1.5 μg/mL rif—DMSO and 1.5 μg/mL rif −7.8 μg/mL rif OD 0.25 in 20 mL Pioreactor vials. Pioreactor cultures were grown for 24 h at 37 °C, with constant stirring at rpm 500. OD600 equiv readings were taken every 5 s.

### Protein Extraction

Msm was grown to an OD of 1.0, treated with 0 and halfxMIC (3.9 μg/mL) rifampicin in triplicate and incubated at 37 °C and harvested at 45 and 180 min post treatment. Similarly, following determination of the sublethal rifampicin concentration, bulk Mtb H37Ra cultures were prepared for subsequent treatments. From this, triplicate cultures were treated with 0xMIC and halfxMIC (0.006 μg/mL) rifampicin for 2 h at 37 °C.

Following treatment, harvested cultures were centrifuged at 4000 rpm at 4 °C for 10 min. The supernatant was discarded and the pellet resuspended in phosphate-buffered saline (PBS) before being transferred to LoBind Eppendorf Tubes and centrifuged. Pellets were washed an additional two times and stored at −20 °C until further processing.

### Cell Lysis and Protein Digestion

Frozen pellets were thawed on ice, before being lysed by trifluoroacetic acid (TFA). Briefly, lysis involved adding the minimum amount of LC/MS grade TFA ≥99% (Sigma-Aldrich) before quenching with four volumes of 4 M Tris buffer with a postneutralization pH ≈ 8.0. Protein concentrations were then quantified using the Coomassie (Bradford) protein assay.^43^ Five μg of protein per sample was transferred to a 96-well plate for trypsin digestion. Briefly, Dithiothreitol (DTT) was added to a final concentration of 3 mM and incubated for 20 min at RT followed by 15 mM Iodoacetamide (IAA) for 30 min in darkness. Sequence grade trypsin (Pierce MS-Grade) was added at a trypsin to protein ratio of 1:100 and allowed to digest overnight at 37 °C.

### Liquid Chromatography-Tandem Mass Spectrometry

200 ng of each sample’s peptide digests were desalted and loaded onto EvoTips (Evosep Biosystems) following the manufacturers protocol (EV2011). The peptides were loaded onto an Evosep One LC and separated using the 60 samples per day (SPD) Evosep method and analyzed on a timsTOF Pro2 mass spectrometer (Bruker Daltonics), using data-independent acquisition (DIA) and parallel accumulation serial fragmentation (PASEF) methodology.^44^ The dia-PASEF window parameters were as follows: Mass width −25.00 Da; Mass overlap −0.00 Da; Mass steps per cycle −21; Mobility overlap −0.00 1/K0 and No. mobility windows −1. Additionally, a mass range of 475 to 1000 Da and mobility range of 0.85 to 1.27 1/K0 were used. The analysis used a PepSep C18 8 cm ID 150 1.5 μm column (Bruker Daltonics).

### Database Search and Statistical Analysis

The Msm (UniProt proteome ID: UP000000757) and Mtb H37Rv (UniProt proteome ID: UP000001584) reference proteomes,^45^ were used for library free analysis in DIA-NN version 1.9.^46,47^ Missed cleavages was set to 1, with 0 variable modifications and carbamidomethylation of cysteine set as a fixed modification. The remaining specifications included peptide length of 7–30 amino acids, precursor charge range of 2–3, precursor m/z range of 300–1300 and fragment ion m/z range of 100–1700 using an MS mass accuracy of 20 ppm, with match-between-runs (MBR), no shared spectra and heuristic protein inference and double-pass mode enabled. Statistical analyses were performed using Perseus,^48^ MetaboAnalyst,^49^ R 4.4.2 ^50^ and Python 3.14.1. The packages used to generate plots included ggplot, pheatmap, seaborn and matpotlib. Significantly dysregulated proteins between sample groups in Msm were identified using limma (FDR <0.05) differential expression analysis^51,52^ using R 4.4.2 with readxl, limma and openxlsx libraries. In Mtb H37Ra, Metaboanalyst was used to perform *t* tests (FDR <0.05) and calculate fold-change. Following statistical analyses, UniProt^45^ and Mycobrowser^53^ were used to retrieve the ID’s of significantly dysregulated proteins. STRING^54^ was then used to generate protein–protein interaction networks and functional enrichment analyses. Enrichments were then clustered using Markov Cluster (MCL) algorithm to find natural clusters based on the stochastic flow and visualized in Cytoscape.^55^

## Supplementary Material



## Data Availability

The mass spectrometry proteomics data have been deposited to the ProteomeXchange Consortium via the PRIDE^56^ partner repository with the data set identifier PXD075873.

## Notes

Supporting Information Figures S1–S4 and supplementary Table S1 with graphical representations of microbroth dilution MIC’s, growth curves and images of Msm colonies grown on solid media (PDF)
