# Supplementary material for: Beyond Inhibition: Sublethal Rifampicin-Induced Molecular Adaptations Confer Phenotypic Drug Tolerance in Mycobacteria
Source: ACS Infect Dis. 2026 Apr 28;12(5):1600–10. doi: 10.1021/acsinfecdis.5c00701 (PMC13162311; doi:10.1021/acsinfecdis.5c00701)
Supplement: Supplementary file 1 [file id5c00701_si_001.pdf]

Supporting Information:

Beyond Inhibition: Sub-lethal Rifampicin-Induced Molecular Adaptations Confer Phenotypic Drug Tolerance in Mycobacteria

Blake D. Stuart[†], Anja van der Merwe[†], Melissa D. Chengalroyen[‡], Atica Moosa[‡], Digby F. Warner[‡,§], Jonathan M. Blackburn[†,§], *Tariq A. Ganief [†,§]

†. Division of Chemical and Systems Biology, Department of Integrative Biomedical Sciences, Faculty of Health Sciences, University of Cape Town, Cape Town 7925, South Africa

‡. Molecular Mycobacteriology Research Unit, Division of Medical Microbiology, Department of Pathology, Faculty of Health Sciences, University of Cape Town, Cape Town 7925, South Africa

§. Institute of Infectious Disease and Molecular Medicine, Faculty of Health Sciences, University of Cape Town, Cape Town 7925, South Africa

*Email: tariq.ganief@uct.ac.za

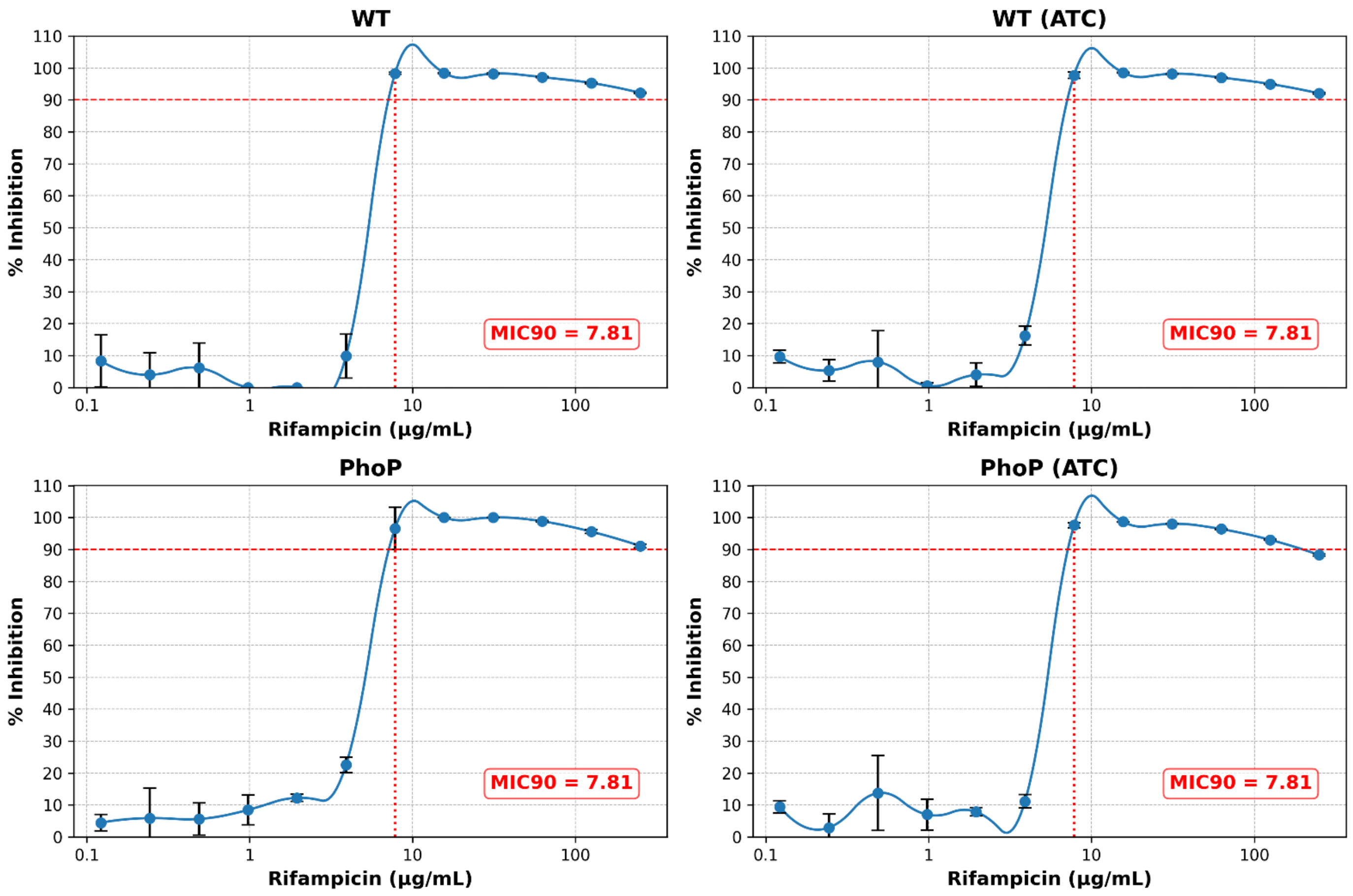

Figure S1: % growth inhibition curves showing the minimum inhibitory concentrations (MIC) of Msm to rifampicin. Strains shown include WT and PhoP CRISPRi hypomorph (containing a pIRL117 CRISPRi plasmid). MIC's were tested in the presence and absence of anhydrotetracycline (ATc) which is the conditional inducer of the CRISPRi knockdown.

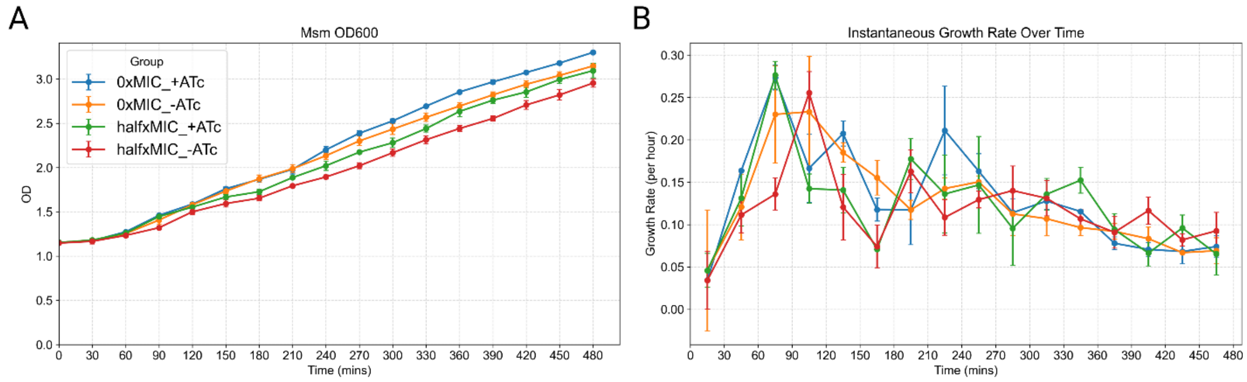

Figure S2: Msm growth curves in response to sub-lethal rifampicin treatment in the presence and absence of ATc. **(A)** OD600 readings against time and **(B)** growth rate showing change in OD per hour for untreated and halfxMIC treated samples in the presence and absence of ATc.

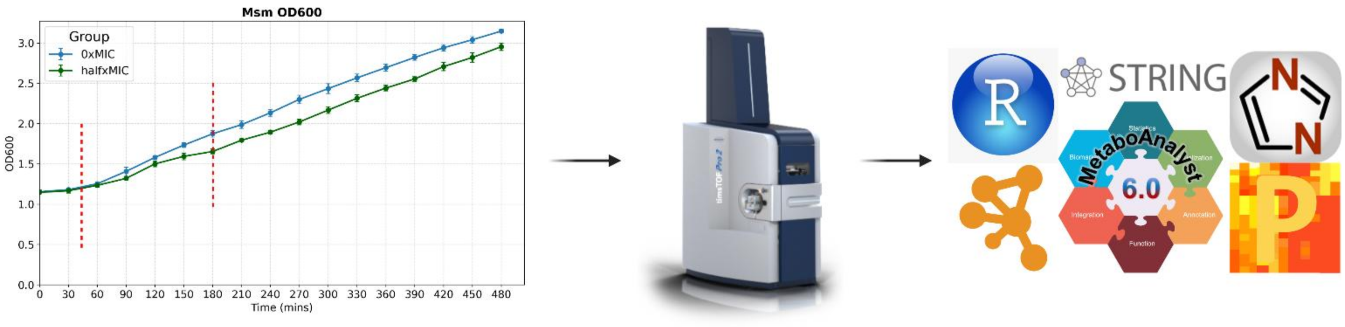

Figure S3: Graphical overview of the methods utilised in this study, demonstrating the cell culture, proteomic and data analysis steps.

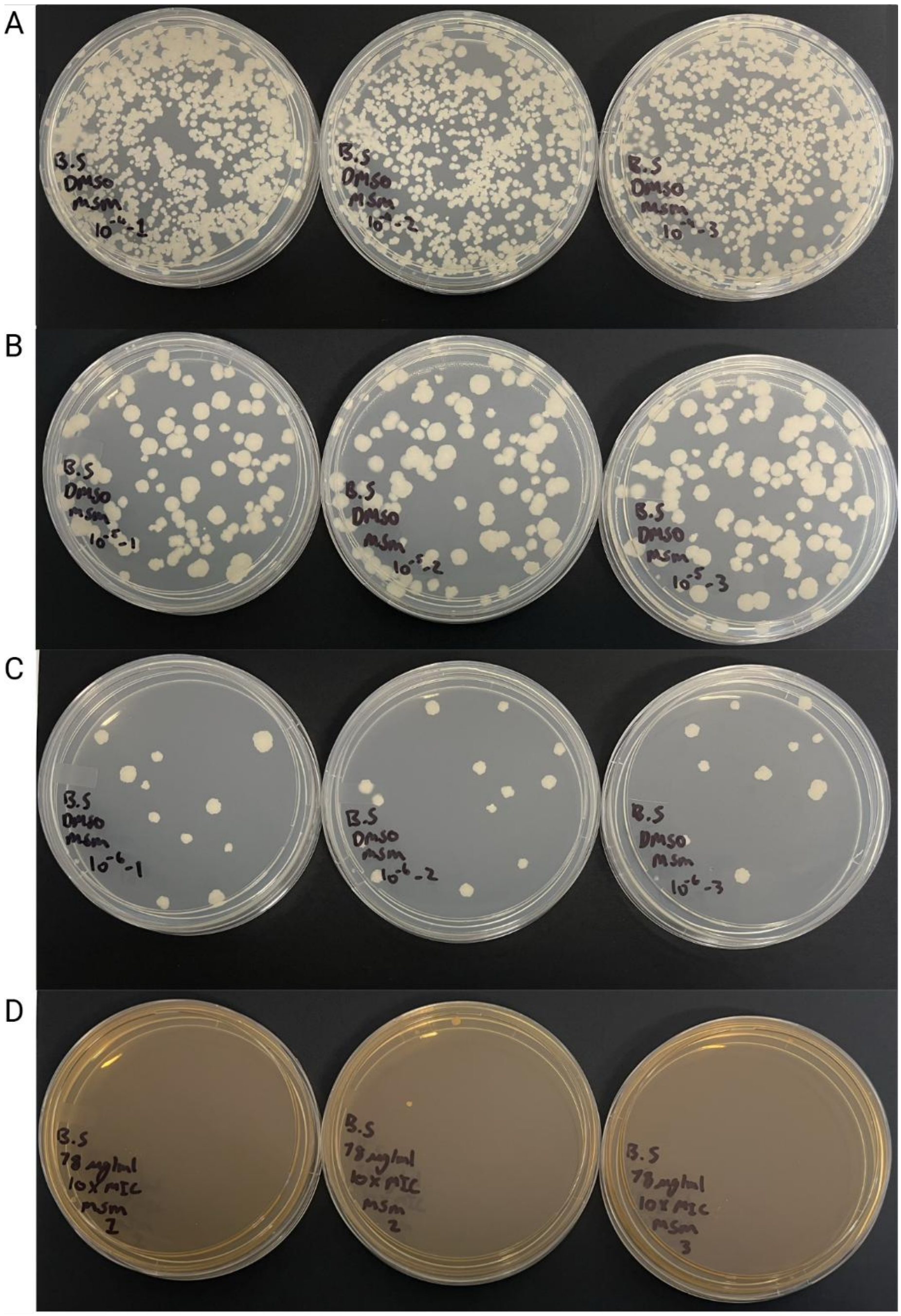

Figure S4: Phenotypic tolerance acquisition experiment of Msm plated on 7H10 OADC plates. Msm was grown in bulk in 7H9 OADC media before being normalised to OD 0.3 and pretreated with 1.5 µg/mL rifampicin for 7 h. After pretreatment 1 mL of culture was spun down, washed twice with fresh 7H9 media and then serially 10-fold diluted – 100 mL in 900 mL 7H9 media. 100 µL of culture was plated from 10^{-4}, 10^{-5} and 10^{-6} dilutions on DMSO (0.2 % v/v) control plates and 100 µL of undiluted culture was plated on 78 µg/mL rifampicin plates (10xMIC). All plates were prepared in triplicate and incubated at 37 °C for 4 days. **(A)** 3x DMSO control plates on which 100 µL of 10^{-4} dilution stock was plated – estimated 1100 cells. **(B)** 3x DMSO control plates on which 100 µL of 10^{-5} dilution stock was plated – average 110 cells. **(C)** 3x DMSO control plates on which 100 µL of 10^{-6} dilution stock was plated – average 11 cells. **(D)** 3x 78 µg/mL (10xMIC) rifampicin plates on which 100 µL of undiluted Msm was plated – and from colony counts of DMSO controls, an estimated 11,000,000 cells were plated resulting in 1 detectable colony.

Table S1: Results of microbroth dilution MIC determination in Mtb H37Ra.

**Concentration of rifampicin (µg/mL)**	**Growth (+/-)**
**0.64**	-
**0.32**	-
**0.16**	-
**0.08**	-
**0.04**	-
**0.02**	-
**0.01**	-
**0.005**	-
**0.0025**	-
**0.0012**	-
**0.0006**	+
**0.0003**	+
